# Systemic Inflammation Indices, Chemokines, and Metabolic Markers in Perimenopausal Women

**DOI:** 10.3390/nu17172885

**Published:** 2025-09-06

**Authors:** Anna Maria Cybulska, Kamila Rachubińska, Elżbieta Grochans, Mateusz Bosiacki, Donata Simińska, Jan Korbecki, Anna Lubkowska, Mariusz Panczyk, Magdalena Kuczyńska, Daria Schneider-Matyka

**Affiliations:** 1 Department of Nursing, Pomeranian Medical University in Szczecin, Żołnierska 48 St., 71-210 Szczecin, Poland; anna.cybulska@pum.edu.pl (A.M.C.); kamila.rachubinska@pum.edu.pl (K.R.); elzbieta.grochans@pum.edu.pl (E.G.); daria.schneider.matyka@pum.edu.pl (D.S.-M.); 2 Department of Biochemistry and Medical Chemistry, Pomeranian Medical University in Szczecin, Powstańców Wielkopolskich. 72 St., 70-111 Szczecin, Poland; donata.siminska@pum.edu.pl; 3 Department of Anatomy and Histology, Collegium Medicum, University of Zielona Góra, Zyty 28, 65-046 Zielona Góra, Poland; jan.korbecki@pum.edu.pl; 4 Department of Functional Diagnostics and Physical Medicine, Pomeranian Medical University in Szczecin, 71-210 Szczecin, Poland; anna.lubkowska@pum.edu.pl; 5 Department of Education and Research in Health Sciences, Faculty of Health Sciences, Medical University of Warsaw, 00-635 Warsaw, Poland; mariusz.panczyk@wum.edu.pl; 6 Subdepartment of Long-Term Care and Palliative Medicine, Department of Social Medicine, Faculty of Health Sciences, Pomeranian Medical University in Szczecin, 48 Żołnierska St., 71-210 Szczecin, Poland; magdalena.kuczynska@pum.edu.pl

**Keywords:** chemokines, systemic inflammation, SII, SIRI, metabolic syndrome, perimenopause

## Abstract

**Background:** Menopause and metabolic syndrome (MetS) are linked to chronic low-grade inflammation. However, the role of chemokines and systemic inflammatory indices such as the systemic immune-inflammation index (SII) and systemic inflammatory response index (SIRI) in perimenopausal women remains poorly understood. **Methods:** This cross-sectional study evaluated inflammatory markers, chemokines, and systemic indices in perimenopausal women recruited in Poland. Sociodemographic and health-related information was obtained using a custom questionnaire, along with anthropometric measurements and laboratory analyses. **Results:** A total of 230 women aged 44–65 years were included. Women with BMI ≥ 25 kg/m^2^ had significantly higher IL-6 (median 4.9 vs. 2.3 pg/mL, *p* < 0.01) and CRP levels (3.8 vs. 1.6 mg/L, *p* < 0.05), as well as increased HOMA-IR (2.6 vs. 1.5, *p* < 0.01), compared with those with normal BMI. Positive correlations were found between SII and CXCL5 (r = 0.21, *p* = 0.01), and between SIRI and CXCL2 (r = 0.19, *p* = 0.02), CXCL5 (r = 0.23, *p* = 0.01), and CXCL9 (r = 0.24, *p* = 0.01). **Conclusions:** Excess body weight in perimenopausal women was associated with elevated IL-6, CRP, and insulin resistance, together with BMI-dependent correlations of chemokines with SII and SIRI. These findings highlight the potential of SII and SIRI as accessible screening tools for identifying women at risk of MetS. Future longitudinal studies are needed to confirm their predictive value and clinical applicability.

## 1. Introduction

Menopause marks the permanent cessation of menstrual cycles, usually between 45 and 55 years of age [1]. The associated decline in estrogen contributes to bone loss, central fat accumulation, and heightened cardiovascular risk. This transition also predisposes women to type 2 diabetes and metabolic syndrome (MetS) [2].

Metabolic syndrome (MetS) is a widespread health concern, affecting up to one-third of adults in certain populations [3,4,5,6]. It has been described by several expert groups, including NCEP ATP III and the International Diabetes Federation, and harmonized diagnostic criteria have since been proposed [3,4,5,6]. These definitions emphasize the coexistence of cardiometabolic disturbances—obesity, dyslipidemia, hyperglycemia, and hypertension—that are closely associated with sedentary lifestyle, poor dietary patterns, and chronic stress, ultimately fostering inflammation and oxidative imbalance [7,8,9,10,11].

The mechanisms underlying MetS remain incompletely clarified, and it is still debated whether its components represent distinct disorders or facets of a single pathological process [12]. Visceral fat is considered a major contributor, acting as a metabolic trigger that activates multiple harmful pathways [13]. Central features such as insulin resistance, persistent low-grade inflammation, and neurohormonal imbalance are regarded as key drivers that elevate the risk of type 2 diabetes and cardiovascular disease [14]. The chronic inflammatory state associated with MetS is of particular interest, as it involves immune-mediated activation of multiple inflammatory cascades, including elevated secretion of proinflammatory cytokines and chemokines. These mediators can adversely affect vascular endothelium and immune system regulation, further promoting metabolic and cardiovascular complications.

In this context, growing attention is being paid to novel inflammatory biomarkers that offer insight into systemic immune status. One such marker is the Systemic Immune-Inflammation Index (SII), a recently introduced parameter calculated from neutrophil, lymphocyte, and platelet counts. SII reflects the balance between host inflammatory and immune responses and has emerged as a potential indicator of subclinical inflammation in metabolic and cardiovascular disorders. The systemic inflammatory response index (SIRI) is a composite indicator used to assess systemic inflammation and immune function, based on the ratios of neutrophils, monocytes, and lymphocytes in peripheral blood. These indices may be useful in evaluating the risk and prognosis of inflammation-related diseases [15,16,17]. Previous studies have shown that SII and SIRI may serve as independent prognostic markers in cancer [18], rheumatoid arthritis [19], hepatic steatosis [20], diabetes [21], hyperlipidemia [17], acute ischemic stroke [22], cardiovascular risk [23], and metabolic syndrome [24].

Chemokines, as chemotactic cytokines, direct the migration of immune cells [25,26] and are essential for inflammatory responses and immune system function. Immune cells express a variety of chemokine receptors [27], and each chemokine regulates the migration and function of specific immune cell subsets [28]. Chemokines also exert broader biological effects unrelated to the immune system [29,30,31]. In this study, we analyzed the concentrations of CXCL1, CXCL2, CXCL3, and CXCL5 (GRO chemokines), which are ligands for the CXCR2 receptor [32] and chemoattractants for neutrophils and basophils [27,33], as well as CXCL9 (MIG), a ligand for the CXCR3 receptor and a chemoattractant for dendritic cells and T cells [27], which play key roles in immune regulation.

Indoleamine 2,3-dioxygenase (IDO) catalyzes the conversion of tryptophan to kynurenine, a metabolic route often upregulated during inflammation. Enhanced IDO activity depletes tryptophan while increasing kynurenine, potentially influencing serotonin metabolism. Although research directly connecting SII with this pathway is scarce, inflammation has been consistently associated with elevated IDO activity [34].

Key inflammatory mediators—including IL-1β, TNF-α, IFN-γ, IL-6, IL-1α, and CRP—are crucial for the initiation and regulation of immune responses, and their overproduction is closely linked to metabolic and cardiovascular disturbances [35,36,37,38]. Adipokines, including adropin and adiponectin, are important modulators of energy balance and inflammatory pathways, and are often studied in relation to metabolic syndrome [39,40,41]. Another adipokine, visfatin (also referred to as NAMPT or PBEF), has been implicated in immune and inflammatory responses [42].

In conclusion, although direct studies examining the association between SII and SIRI values, chemokine concentrations, and BMI are limited, there is indirect evidence supporting a link through their role in regulating energy metabolism and inflammatory pathways. To date, no studies have directly analyzed the relationship between SII and SIRI values and inflammation, metabolic syndrome, and its components in menopausal women.

Previous research on SII and SIRI has predominantly focused on oncology, cardiovascular disease, and diabetes, while studies addressing these indices in women undergoing the menopausal transition are lacking. This gap may stem from the heterogeneity of this population with respect to age, hormonal status, and comorbidities, which complicates recruitment and analysis. By examining BMI-dependent associations of chemokines with SII and SIRI in perimenopausal women, our study provides novel insights that extend current knowledge on systemic inflammation and its relevance to metabolic syndrome.

Although menopause and metabolic syndrome have been widely studied in the context of inflammation, the specific relationships between chemokines and novel systemic indices such as SII and SIRI remain largely unexplored. To our knowledge, no previous research has investigated these associations in perimenopausal women, a group particularly vulnerable to metabolic and inflammatory disturbances. Addressing this gap may provide new insights into the role of BMI-dependent immune mechanisms in the menopausal transition. To our knowledge, this is the first study to investigate BMI-dependent associations between systemic inflammatory indices (SII, SIRI) and chemokine concentrations in perimenopausal women. While SII and SIRI have been extensively studied in oncology and cardiometabolic disorders, their relationship with immune–metabolic alterations during the menopausal transition remains unexplored. By addressing this gap, our study contributes novel evidence with international relevance, highlighting potential pathways linking systemic inflammation, chemokines, and metabolic risk in this vulnerable population.

Therefore, the aim of this study was to evaluate the associations between systemic inflammatory indices (SII, SIRI), inflammatory markers, and chemokine levels in perimenopausal women, with particular attention to differences according to body mass index.

## 2. Materials and Methods

### 2.1. Recruitment Process

Three hundred women between the ages of 45 and 65 residing in the West Pomeranian Voivodeship were invited to take part in the study. Eligibility criteria included age between 44 and 65 years, absence of menopausal hormone therapy (MHT) use, no prior psychiatric treatment, a typical diet based on traditional Polish cuisine, and no current use of dietary supplements. Dietary habits were self-reported by the participants. Women were eligible if they declared adherence to a traditional Polish diet, which is typically rich in meat, potatoes, bread, and dairy products. No validated dietary recall or food frequency questionnaire was administered, which should be considered a limitation of the study. Participants were excluded if they had a history of or were undergoing MHT, had received psychiatric treatment, had been diagnosed with thyroid disorders or cancer, declined participation, or submitted incomplete study documentation. Exclusion criteria were applied to reduce the influence of potential confounding factors. Women receiving menopausal hormone therapy (MHT) were excluded due to its well-documented effects on metabolic and inflammatory processes. Those with a history of psychiatric treatment were not included because psychotropic medications may alter appetite regulation, body weight, and systemic inflammation. Thyroid disorders and cancer were also considered exclusion criteria as they strongly affect metabolic homeostasis and inflammatory status. Finally, the use of dietary supplements was restricted to avoid interference with biochemical and inflammatory markers. While these criteria increased the homogeneity of the study group, they may also limit the generalizability of our findings to the broader population of perimenopausal women.

Sample size estimation was based on demographic data for women aged 45–65 in the West Pomeranian Voivodeship as of 2022, using a 95% confidence level, a 5% margin of error, and an assumed proportion of 0.5. This calculation indicated a minimum required sample size of approximately 350 participants; our final sample of 230 women was slightly below this threshold but comparable to previous studies in this field and sufficient for the planned analyses.

The study adhered to the principles of the Declaration of Helsinki and was approved by the Bioethics Committee of the Pomeranian Medical University in Szczecin (approval no. KB-0012/181/13, issued on 16 December 2013, with an annex dated 18 December 2024). Recruitment was conducted through informational posters in public spaces and advertisements in regional newspapers. All participants provided written informed consent. As participation was voluntary and based on public advertisements, the study sample may not be fully representative of all perimenopausal women in the general population, and selection bias cannot be excluded. This research is part of a broader project evaluating the health status of perimenopausal women in the region.

### 2.2. Research Project

The research was carried out in multiple phases. Following informed consent, participants completed a custom questionnaire collecting sociodemographic data such as age, marital status, residence, educational background, and employment. Menopausal status was categorized as perimenopausal (defined by irregular menstrual cycles indicating menopausal transition) or postmenopausal (absence of menstruation for at least 12 months). In the subsequent stage, fasting blood samples were drawn for immunological and biochemical analyses.

### 2.3. Blood Collection and Biochemical Analysis

Fasting venous blood was collected between 7:00 and 9:30 a.m. after participants rested in a seated position for 10 min. Trained nurses performed the procedures using the Vacutainer system. Collection, storage, and transport followed standardized protocols for handling biological specimens. Biochemical analyses were conducted at a certified laboratory at the Pomeranian Medical University in Szczecin using validated commercial assays. The measured parameters included insulin, glucose, glycated hemoglobin (HbA1c), total cholesterol (TCh), HDL cholesterol, LDL cholesterol, triglycerides (TG), and C-reactive protein (CRP).

Serum concentrations of IL-6, TNF-α, and adiponectin were determined by enzyme-linked immunosorbent assay (ELISA) using kits from Immundiagnostik (Bensheim, Germany). CRP was analyzed by immunonephelometry (Behring Nephelometer II, Dade Behring, Marburg, Germany).

### 2.4. Inflammatory Marker Determination: IL-1α, IL-6, IL-1β, TNF-α, and IFN-γ

For inflammatory marker analysis, blood was collected into serum separation tubes. Concentrations of IL-1α, IL-1β, IL-6, TNF-α, and IFN-γ in serum were measured using ELISA kits from DRG (Marburg, Germany). The following assay parameters were reported:

- IL-1α: sensitivity 1.1 pg/mL; intra-assay CV < 5.4%, inter-assay CV < 10%
- IL-6: sensitivity 2 pg/mL; intra-assay CV 4.2%, inter-assay CV 4.4%
- IL-1β: sensitivity 0.35 pg/mL; intra-assay CV 2.3%, inter-assay CV 4.9%
- IFN-γ: sensitivity 0.03 IU/mL; intra-assay CV 3.2%, inter-assay CV 5.8%
- TNF-α: sensitivity 0.7 pg/mL

Serum samples were diluted based on manufacturer instructions and pilot test results to ensure optimal detection. Protein concentrations were measured using ELISA kits (FineTest, Wuhan, China) for the following chemokines:

- CXCL1 (GROα): EH0005; range 15.625–1000 pg/mL; sensitivity 9.375 pg/mL
- CXCL3 (GROβ): EH3178; range 15.625–1000 pg/mL; sensitivity 9.375 pg/mL
- CXCL2 (GROγ): EH3179; range 7.813–500 pg/mL; sensitivity 4.688 pg/mL
- CXCL5 (ENA-78): EH0106; range 31.25–2000 pg/mL; sensitivity 18.75 pg/mL
- CXCL9 (MIG): EH0008; range 31.25–2000 pg/mL; sensitivity 18.75 pg/mL

All assays were conducted in line with the manufacturers’ protocols. Absorbance was measured using a BiochromAsys UVM 340 microplate reader (Biochrom Ltd., Cambridge, UK).

### 2.5. Calculation of Inflammatory Indices: SII and SIRI

The Systemic Immune-Inflammation Index (SII) was computed as follows [24]:

SII = (Platelet Count × Neutrophil Count)/Lymphocyte Count

The Systemic Inflammatory Response Index (SIRI) was calculated as follows:

- SIRI = (Neutrophil Count × Monocyte Count)/Lymphocyte Count
- Reference values were adopted from the literature [24]:
- Low SII: 1.52 ≤ SII < 314.93
- Moderate SII: 314.93 ≤ SII < 626.51
- High SII: 626.51 ≤ SII < 8486

### 2.6. Statistical Analysis

To ensure the validity of parametric analyses, all continuous variables were assessed for normality using the Anderson–Darling test. When deviations from normality were identified, Box–Cox transformations were applied to stabilize variances and approximate Gaussian distributions, thereby fulfilling the assumptions of linearity and homoscedasticity required for Pearson’s correlation coefficient (r). This approach was adopted to improve the interpretability and statistical power of correlation analyses.

To reduce the risk of false positives due to multiple testing in correlation matrices, the false discovery rate (FDR) was controlled using the Benjamini–Hochberg procedure, with an adjusted *q*-value < 0.05 considered statistically significant. This correction was applied exclusively to correlation analyses, and not to group comparisons.

Group comparisons between participants with BMI < 25 kg/m^2^ and those with BMI ≥ 25 kg/m^2^ were performed using the Brunner–Munzel test, a robust nonparametric method appropriate for data with unequal variances and non-normal distributions. Descriptive statistics were reported as means with standard deviations, medians with interquartile ranges, and minimum–maximum values. All statistical analyses were conducted using IBM SPSS Statistics, version 29 and jamovi (Version 2.5) [Computer Software]. A two-tailed *p*-value of <0.05 was considered statistically significant for individual tests without multiplicity correction.

## 3. Results

### 3.1. Participant Characteristics

The study sample consisted of 230 women, the majority of whom were postmenopausal (63%). The mean age was 52.9 ± 5.05 years (range: 44–66). Most participants (79%) lived in cities with populations exceeding 100,000. A substantial proportion were in formal relationships (71.10%), and 90.40% were employed.

Blood samples were analyzed to determine selected hematological and biochemical parameters, chemokine concentrations, and inflammation markers. The mean values of these indicators in the study group were generally within reference ranges; however, in some cases, individual values fell below or exceeded normal limits (Table S1). Detailed characteristics of hematological and biochemical parameters are presented in Table 1 and Table 2.

Women with BMI ≥ 25 kg/m^2^ exhibited significantly higher fasting glucose, insulin, and HOMA-IR values, along with elevated serum IL-6 and CRP concentrations compared with women of normal weight. Furthermore, BMI-dependent correlations were observed between systemic inflammatory indices and selected chemokines: SII and SIRI correlated positively with CXCL2, CXCL5, and CXCL9. These findings highlight the interplay between excess body weight, systemic inflammation, and chemokine activity in perimenopausal women (Table 1).

In addition, statistically significant differences were observed in glucose concentration [mg/dL], HbA1c [%], insulin levels [µIU/mL], HOMA-IR, indoleamine 2,3-dioxygenase [ng/mL], triglycerides (TG) [mg/dL], kynurenine [ng/mL], QUICKI, and HDL cholesterol [mg/dL] between the BMI groups. Overweight and obese participants exhibited significantly higher levels of glucose, HbA1c, insulin, HOMA-IR, indoleamine 2,3-dioxygenase, triglycerides, and kynurenine compared to participants with normal body weight. Conversely, women with a BMI < 25 kg/m^2^ had significantly higher QUICKI and HDL cholesterol levels than those with higher BMI values (Table 2).

### 3.2. Inflammatory Markers

No significant differences were observed for IL-1β, TNF-α, IFN-γ or most chemokines across BMI groups; detailed data are available in Supplementary Tables S2–S4.

**Figure 1:**
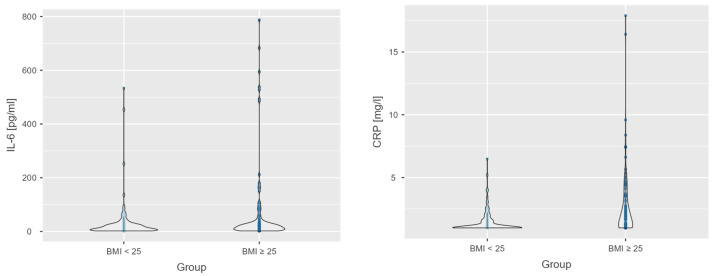
Violin plots presenting the distribution of inflammatory markers (IL-6 and CRP) across BMI categories (<25 vs. ≥25).

Additionally, Table S3 presents a comparison of inflammatory markers and indices between individuals with BMI < 25 and those with BMI ≥ 25. Higher mean levels of IL-6 and CRP were observed in the BMI ≥ 25 group, indicating an elevated inflammatory response in individuals with overweight or obesity. For example, the mean IL-6 concentration was 31.23 pg/mL in the BMI < 25 group and 60.06 pg/mL in the BMI ≥ 25 group. Similarly, CRP levels were substantially higher in the BMI ≥ 25 group (2.56 mg/L) compared to the BMI < 25 group (1.44 mg/L), suggesting a more pronounced systemic inflammatory state in individuals with higher BMI (Table S3). These differences are also illustrated in Figure 1.

In summary, the findings highlight a clear association between elevated BMI and increased levels of IL-6 and CRP, underscoring the role of excess body weight in promoting systemic inflammation.

### 3.3. Chemokine Levels and Correlations

Correlation analysis between chemokines (CXCL1, CXCL2, CXCL3, CXCL5, CXCL9), proinflammatory cytokines (IL-1β, TNF-α, IFN-γ, IL-6, IL-1α), and inflammatory indices (CRP, SII, SIRI) revealed several noteworthy associations that differed according to body mass index (BMI).

**Figure 2:**
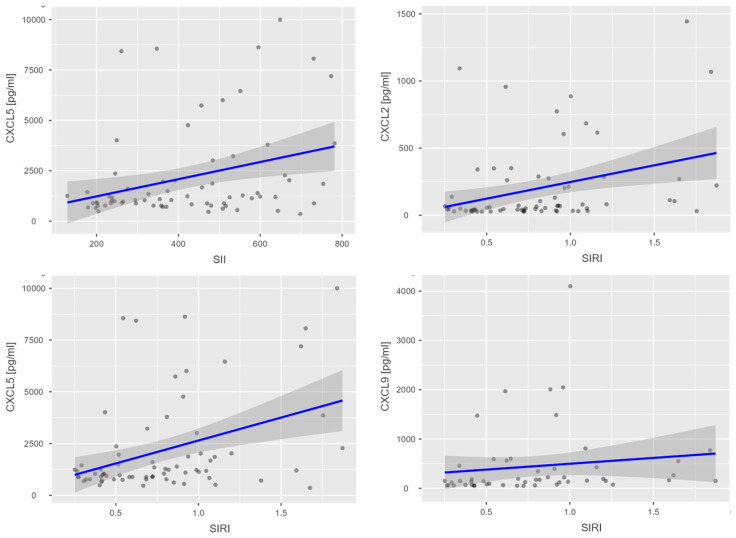
Scatterplots illustrating correlations between systemic inflammatory indices (SII and SIRI) and selected chemokines (CXCL2, CXCL5, and CXCL9) in women with BMI < 25. Black dots represent individual observations, the blue line indicates the fitted linear regression, and the grey shaded area denotes the 95% confidence interval.

Among women with a BMI < 25 kg/m^2^, a statistically significant positive correlation was observed between SIRI and CXCL2 (r = 0.335, *p* = 0.005), suggesting a potential association between this inflammatory index and chemokine activity in individuals with normal weight. Additionally, significant positive correlations were identified between SII and CXCL5 (r = 0.319, *p* = 0.007), and between SIRI and CXCL5 (r = 0.388, *p* < 0.001), indicating a possible role of CXCL5 in systemic inflammation in this subgroup. A significant positive correlation was also noted between SIRI and CXCL9 (r = 0.267, *p* = 0.023) among classical inflammatory markers (Table S4). These associations are further illustrated in Figure 2, which presents scatterplots of SII with CXCL5 and of SIRI with CXCL2, CXCL5, and CXCL9.

In the overweight and obese group (BMI ≥ 25 kg/m^2^), a significant correlation was found between IL-1α and CXCL9 (r = 0.387, *p* = 0.001), suggesting BMI-dependent interactions between chemokines and cytokines.

These results suggest that selected chemokine–inflammatory marker relationships are modulated by BMI, with CXCL2, CXCL5, and CXCL9 emerging as potential mediators of inflammation in women, particularly in relation to SIRI and SII (Table S4).

**Figure 3:**
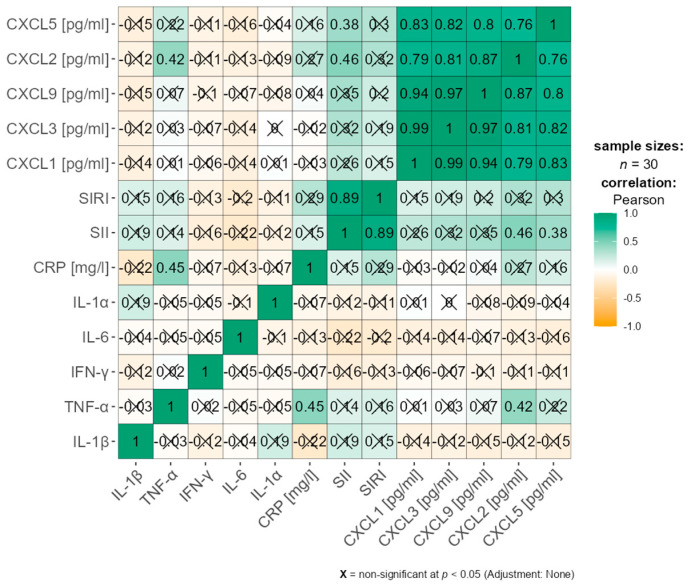
Heatmap presenting Pearson correlation coefficients between chemokines and inflammatory markers (CRP, SII, SIRI) in women with BMI < 25.

**Figure 4:**
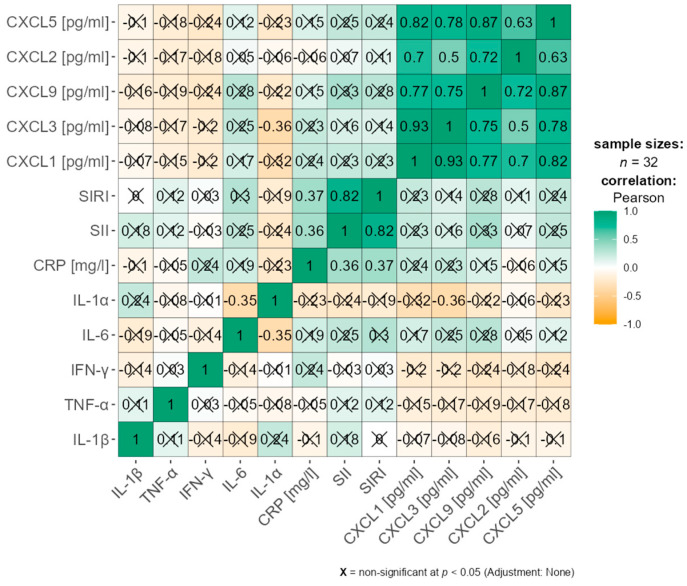
Heatmap presenting Pearson correlation coefficients between chemokines and inflammatory markers (CRP, SII, SIRI) in women with BMI ≥ 25.

To improve clarity, these correlations are also visualized in a heatmap (Figure 3 and Figure 4), while detailed statistical values remain available in Supplementary Table S4.

Table 3 presents the correlation coefficients between selected chemokines (CXCL1, CXCL2, CXCL3, CXCL5, CXCL9) and metabolic/inflammatory biomarkers—including adropin, adiponectin, indoleamine 2,3-dioxygenase (IDO), tryptophan, kynurenine, serotonin, and visfatin—stratified by BMI category (<25 vs. ≥25).

Among women with a BMI < 25 kg/m^2^, a statistically significant negative correlation was found between adiponectin and CXCL2 (r = −0.27, *p* = 0.024), indicating a possible inverse relationship between this anti-inflammatory adipokine and chemokine expression. In addition, adropin was positively associated with CXCL3 (r = 0.259, *p* = 0.01), and kynurenine also showed a significant positive correlation with CXCL3 (r = 0.236, *p* = 0.02), suggesting potential interactions between metabolic and inflammatory pathways in individuals with normal body weight. No statistically significant correlations were observed between the chemokines and tryptophan, serotonin, IDO activity, or visfatin in this subgroup (Table 3).

In contrast, in women with a BMI ≥ 25 kg/m^2^, tryptophan was positively correlated with CXCL2 (r = 0.25, *p* = 0.003), while IDO activity (r = −0.25, *p* = 0.034) and visfatin (r = −0.243, *p* = 0.04) both demonstrated significant negative correlations with CXCL9. No other significant associations were detected between the chemokines and adropin, adiponectin, kynurenine, or serotonin in this BMI group.

These results indicate BMI-dependent variation in the associations between chemokines and metabolic or inflammatory markers, pointing to distinct regulatory pathways in normal-weight versus overweight and obese women (Table 3).

We analyzed the associations between chemokine levels (CXCL1, CXCL2, CXCL3, CXCL5, and CXCL9) and various hematological and biochemical blood parameters in women. We examined the relationships between circulating chemokine levels (CXCL1, CXCL2, CXCL3, CXCL5, and CXCL9) and selected hematological and biochemical parameters in women. CXCL1 levels showed weak but statistically significant negative correlations with several red blood cell indices: hemoglobin (Hb) (r = −0.157, *p* = 0.018), mean corpuscular volume (MCV) (r = −0.188, *p* = 0.004), mean corpuscular hemoglobin (MCH) (r = −0.201, *p* = 0.002), and mean corpuscular hemoglobin concentration (MCHC) (r = −0.165, *p* = 0.013). These findings suggest that lower CXCL1 concentrations may be associated with higher values of these erythrocyte-related markers. No other significant associations were observed between CXCL1 and the remaining hematological or biochemical parameters (Table S5).

For CXCL2, a weak negative correlation was noted with lymphocyte percentage (r = −0.171, *p* = 0.040) and HbA1c levels (r = −0.168, *p* = 0.043), while a weak positive correlation was found with neutrophil count (r = 0.179, *p* = 0.032). No additional statistically significant correlations were observed for CXCL2.

CXCL3 was weakly negatively correlated with MCH (r = −0.133, *p* = 0.048) and MCV (r = −0.134, *p* = 0.047), but no other hematological or biochemical variables showed significant associations.

A statistically significant weak negative correlation was observed between CXCL5 levels and basophil percentage (r = −0.237, *p* = 0.005), indicating that lower CXCL5 levels may be linked to elevated basophil counts. No further significant correlations were found for this chemokine.

CXCL9 exhibited several statistically significant associations. Weak negative correlations were observed with lymphocyte percentage (r = −0.259, *p* = 0.004), MCH (r = −0.218, *p* = 0.015), and MCV (r = −0.237, *p* = 0.008). In contrast, weak positive correlations were found with total leukocyte count (r = 0.248, *p* = 0.006), erythrocyte count (r = 0.221, *p* = 0.014), neutrophil count (r = 0.307, *p* < 0.001), and leukocyte percentage (r = 0.248, *p* = 0.006). No additional significant associations were identified for CXCL9.

Taken together, these findings reveal weak but meaningful correlations between selected chemokines and hematological markers, indicating possible links between chemokine expression and systemic inflammatory or immune activity (Table S5).

## 4. Discussion

### 4.1. Comparison with Previous Studies

Numerous epidemiological studies have demonstrated that immune response and inflammation are key factors in the development of metabolic disorders and chronic diseases [43]. Our findings also revealed elevated concentrations of proinflammatory chemokines in menopausal women. The levels of CXCL1, CXCL3, and CXCL5 were significantly higher compared to reference values, indicating their proinflammatory activity. Although CXCL2 levels remained within the normal range for most participants, the high maximum values suggest individual variability and also point to a potential role of this chemokine in inflammatory processes. Elevated CXCL9 levels in the serum of menopausal women may further indicate chronic immune system activation.

While our findings confirm previously reported elevations of IL-6 and CRP in overweight and obese women, the novelty of this study lies in the BMI-dependent associations of CXCL2, CXCL5, and CXCL9 with systemic inflammatory indices. These chemokines are key mediators of neutrophil recruitment and inflammatory signaling, suggesting that their interaction with SII and SIRI reflects a broader activation of immune-inflammatory pathways during the menopausal transition. Importantly, this highlights the potential clinical utility of SII and SIRI as accessible markers for identifying women at increased risk of metabolic syndrome, complementing traditional laboratory assessments.

Elevated levels of specific chemokines such as CCL2/MCP-1, CCL3/MIP-1α, CCL5/RANTES, and CXCL8/IL-8 alongside low-grade systemic inflammation, have also been observed in other studies involving postmenopausal women [44]. These findings point to a chronic, low-level inflammatory state induced by menopause or estrogen deficiency, characterized in part by enhanced monocyte adhesion to the arterial endothelium. Importantly, chronic administration of low-dose 17β-estradiol or pharmacological inhibition of the renin-angiotensin system was shown to mitigate this inflammatory response. Supporting these in vivo observations, an in vitro study using human arterial and venous endothelial cells demonstrated that leukocytes from healthy postmenopausal women exhibited greater adhesion to arterial endothelium compared to those from premenopausal women, regardless of the inflammatory stimulus applied. Moreover, the study reported significantly elevated circulating levels of CXCL8/IL-8, CCL2, CCL3, and CCL5, as well as increased expression of the adhesion molecule CD11b on monocytes in postmenopausal women, further highlighting the proinflammatory shift associated with estrogen deficiency.

Zeng et al. observed a positive association between SII and metabolic syndrome (along with its components, such as hypertriglyceridemia, low HDL cholesterol, and hyperglycemia) in a study conducted among U.S. adults [45]. Our study demonstrated significant differences in parameters related to metabolic syndrome in women with BMI > 25, including elevated markers of carbohydrate metabolism (glucose, HbA1c, insulin, HOMA-IR, QUICKI) and lipid metabolism (increased TG, decreased HDL cholesterol) compared to women with BMI ≥ 25. Higher levels of IL-6 and CRP were also observed in women with increased BMI. However, chemokine concentrations and SII and SIRI values did not differ significantly, as they were elevated in all menopausal participants regardless of BMI.

These observations regarding metabolic syndrome parameters in the study group can be explained by the presence of a chronic low-grade inflammatory state, which leads to insulin resistance, considered a key mechanism linking all components of metabolic syndrome [46]. It is known that excessive amounts of free fatty acids and glucose can trigger the release of inflammatory mediators such as TNF-α, arachidonic acid, and leukotrienes, which recruit neutrophils to inflamed tissues and initiate the inflammatory response [47,48,49].

Additionally, metabolic syndrome is characterized by increased neutrophil survival and their chronic accumulation at sites of inflammation, resulting in prolonged cytokine release that promotes insulin resistance [50]. Regulatory T cells have been shown to counteract insulin resistance and atherosclerosis by suppressing proinflammatory T cells and macrophages [51,52]. HDL cholesterol can exert anti-inflammatory effects by modulating cholesterol transport and activating T cells [53]. Platelets, which are often highly activated in metabolic syndrome and type 2 diabetes, contribute to inflammation by releasing small molecules and cytokines [54], as well as by promoting immune cell adhesion and participating in the formation of neutrophil extracellular traps [55]. Some pharmacological agents used in the treatment of metabolic syndrome and its components may also have anti-inflammatory properties [46].

The complexity of the inflammatory response may be better reflected by SII, which depends on the roles of immune cells [56]. Previous studies have demonstrated an inverse association between SII and hyperlipidemia [17,57]. Zeng et al. also observed a correlation between SII and various dyslipidemia subtypes, such as low HDL and hypertriglyceridemia [45]. Notably, SII has been identified as an independent risk factor for dyslipidemia both above and below certain thresholds. Furthermore, SII has been shown to have predictive value for diabetes-related complications [58,59,60] and depression [61]. However, the relationship between SII and hyperglycemia risk remains not fully understood.

Menopause may be associated with weight gain. During this process, adipocyte hypertrophy and proliferation trigger the activation and recruitment of innate immune cells, contributing to the formation of a proinflammatory microenvironment within adipose tissue [62].

In such an environment, increased inflammatory and metabolic demand induces changes in proinflammatory cytokines, adipokines, free fatty acids, and other markers, ultimately disrupting the homeostasis of organs and tissues [63]. An increase in peripheral leukocyte count serves as a marker of inflammation, and alterations in platelet, neutrophil, monocyte, and lymphocyte counts have been linked to obesity. Previous research has shown that neutrophil and platelet counts tend to rise with increasing body weight, while lymphocyte counts exhibit a negative correlation with body weight [64]. In our study, we also observed significant differences in WBC, lymphocyte, and neutrophil counts among women with BMI ≥ 25. However, in normal-weight menopausal women, we noted a wide range of WBC values and high maximum levels, pointing to individual variability.

Monocytes have been identified as an important source of neutrophil chemokines during the progression of obesity. However, comprehensive prognostic markers such as SII and SIRI, which incorporate peripheral platelet, neutrophil, monocyte, and lymphocyte counts, appear to provide a more integrated assessment of systemic inflammation and immune function than single inflammatory markers like CRP. Our findings support the notion that SII and SIRI may not only reflect inflammatory status but also serve as valuable tools in screening menopausal women. Elevated SII levels reflect increased neutrophil and platelet counts and decreased lymphocyte counts, indicating activation of inflammatory processes. Inflammatory states are characterized by enhanced chemokine production, which recruits neutrophils to sites of inflammation. Therefore, higher SII may correlate with elevated chemokine levels, indicating intensified inflammation [65]. SII has been identified as a useful prognostic marker in various inflammatory conditions, and elevated SII levels have been associated with metabolic syndrome [24], hyperlipidemia [66], diabetic nephropathy [67], non-alcoholic fatty liver disease (NAFLD) [68], and obesity [69].

Hormonal changes during menopause affect fat distribution, promoting visceral but not subcutaneous fat accumulation [68,69,70]. Increased visceral adipose tissue is associated with higher production of inflammatory adipokines and cytokines compared to general obesity and carries a greater risk of chronic diseases [71]. In our study, CXCL3 levels in women with BMI < 25 were positively correlated with adropin and kynurenine concentrations, while CXCL2 levels were negatively correlated with adiponectin. Among women with BMI > 25, CXCL9 showed a negative correlation with IDO and visfatin levels and a positive correlation with tryptophan.

Previous research examining the relationship between chronic inflammation markers and abdominal obesity in pre- and postmenopausal women has yielded inconclusive results. Some studies suggest a stronger association between chronic inflammation and abdominal obesity [72], while others did not report similar findings [73]. These discrepancies may stem from variations in selected inflammatory markers [72,73,74] or differences in obesity assessment methods [75].

Earlier research suggests that body composition changes in midlife result from a combination of chronological and reproductive aging processes [76]. Another study indicated that due to aging, BMI increases by approximately 0.14 kg/m^2^ per year in middle-aged women [77]. Similarly, a 2024 meta-analysis by Pernoud et al. among pre- and postmenopausal women showed that the average BMI difference of 0.31 kg/m^2^ between pre- and postmenopausal periods—over an average age difference of 9–10 years—likely reflects chronological aging rather than menopausal status [78].

However, cross-sectional comparisons of inflammatory cytokine levels revealed no significant differences between pre- and postmenopausal women. Abdominal obesity, rather than menopausal status, was identified as the factor associated with chronic low-grade inflammation and proinflammatory cytokine production [79].

In our study, chemokine levels did not differ significantly between women with a BMI > 25 and those with a BMI < 25. However, we observed significant differences in parameters related to carbohydrate and lipid metabolism—key components associated with the risk of metabolic syndrome—as well as in the concentrations of proinflammatory markers, including IL-1β, IL-6, and CRP.

The mechanisms contributing to the increased risk of chronic diseases during the perimenopausal period remain not fully elucidated but may be partly attributed to unfavorable shifts in body composition and persistent low-grade inflammation. In this context, systemic inflammatory indices such as the Systemic Immune-Inflammation Index (SII) and the Systemic Inflammation Response Index (SIRI) may serve not only as indicators of inflammatory status but also as valuable screening tools for identifying perimenopausal women at increased risk of metabolic syndrome and insulin resistance-related conditions.

### 4.2. Novel Contributions

The most novel finding of this study is the BMI-dependent association of CXCL2, CXCL5, and CXCL9 with systemic inflammatory indices (SII and SIRI). These chemokines are critical mediators of neutrophil activation and recruitment, and their correlation with SII and SIRI suggests that simple blood count–derived indices can capture aspects of immune-inflammatory activation in perimenopausal women. Importantly, because SII and SIRI are calculated from routine hematological parameters, they represent low-cost and widely available tools. Their application in screening programs may facilitate the early identification of women at increased risk of metabolic syndrome and guide preventive strategies in this vulnerable population.

### 4.3. Limitations and Future Directions

This study has several limitations. First, its cross-sectional design precludes conclusions regarding causality. Second, all participants were recruited from a single region (West Pomeranian Voivodeship, Poland), which may limit the generalizability of the findings to broader populations. Third, potential confounding factors such as detailed dietary intake, physical activity, and menopausal stage beyond BMI classification were not fully assessed. Fourth, dietary habits were based on self-reported information without the use of validated assessment tools, which may have introduced recall bias. Finally, the relatively small sample sizes for certain chemokine analyses reduce the statistical power for subgroup comparisons. Despite these limitations, our study provides novel insights into the BMI-dependent associations of chemokines with SII and SIRI in perimenopausal women, and highlights the potential clinical utility of these indices as accessible screening tools. Additionally, because participants were recruited through voluntary responses to public advertisements, the study sample may not fully represent the general population of perimenopausal women, which may limit the generalizability of the findings.

## 5. Conclusions

Perimenopausal women with overweight or obesity (BMI ≥ 25 kg/m^2^) showed significantly higher levels of glucose, insulin resistance indices, IL-6, and CRP, confirming the contribution of excess body weight to low-grade systemic inflammation. While absolute chemokine concentrations did not differ by BMI, BMI-dependent correlations of CXCL2, CXCL5, and CXCL9 with systemic inflammatory indices (SII, SIRI) suggest altered regulatory mechanisms of inflammation in this population. These findings highlight the potential of SII and SIRI as simple, cost-effective screening tools for identifying women at increased risk of metabolic syndrome. Future multicenter longitudinal studies are needed to validate these observations and explore their clinical applicability.

## Figures and Tables

**Table 1:** Characteristics of blood morphological parameters in the studied women according to the body mass index (BMI).

Parameter	BMI < 25 (*n* = 101)			BMI ≥ 25 (*n* = 129)			Brunner-Munzel Test	*df*	*p*
	M ± SD	Me (IQR)	Min–Max	M ± SD	Me (IQR)	Min–Max			
Hemoglobin [g/dL]	13.25 ± 0.88	13.30 (0.90)	9.70–15.00	13.69 ± 1.00	13.70 (1.10)	9.50–16.40	4.287	226.6	<0.001
MCV [fL]	85.95 ± 4.37	86.30 (4.80)	65.10–94.10	85.76 ± 3.92	86.60 (3.70)	67.20–94.50	−0.375	200.1	0.708
MCH [pg]	29.72 ± 1.87	29.90 (1.90)	20.50–32.60	29.67 ± 1.78	30.00 (1.60)	21.30–32.90	−0.335	211.5	0.738
RBC [million/µL]	4.47 ± 0.26	4.45 (0.42)	3.97–5.08	4.62 ± 0.28	4.60 (0.38)	3.98–5.58	4.275	221.6	<0.001
Hematocrit [%]	38.33 ± 2.20	38.20 (2.60)	30.80–43.00	39.63 ± 2.62	39.70 (3.00)	30.50–47.00	4.538	227.2	<0.001
MCHC [g/dL]	34.55 ± 0.82	34.70 (0.90)	30.80–36.20	34.58 ± 0.85	34.60 (1.10)	31.10–36.50	0.097	227.5	0.923
RDW-CV [%]	13.46 ± 0.98	13.30 (0.90)	12.10–18.30	13.51 ± 0.90	13.40 (1.00)	12.00–17.90	0.704	218.4	0.482
WBC [thousand/µL]	5.65 ± 1.39	5.53 (1.60)	3.22–11.98	6.18 ± 1.71	5.91 (2.15)	3.46–11.15	2.24	225.5	0.026
Monocytes [thousand/µL]	0.45 ± 0.13	0.44 (0.16)	0.22–0.89	0.47 ± 0.12	0.47 (0.14)	0.18–0.83	1.415	202.8	0.159
Monocytes [%]	8.08 ± 1.76	7.80 (2.30)	4.80–14.30	7.80 ± 1.72	7.80 (2.40)	3.70–12.70	−0.948	218.2	0.344
Basophils [thousand/µL]	0.03 ± 0.02	0.03 (0.02)	0.01–0.09	0.03 ± 0.02	0.03 (0.02)	0.00–0.11	−0.552	223.8	0.581
Basophils [%]	0.55 ± 0.27	0.50 (0.30)	0.10–1.40	0.52 ± 0.31	0.50 (0.30)	0.00–1.70	−1.645	221.5	0.101
Eosinophils [thousand/µL]	0.14 ± 0.10	0.12 (0.08)	0.01–0.75	0.16 ± 0.10	0.13 (0.11)	0.02–0.65	1.607	216.2	0.11
Eosinophils [%]	2.45 ± 1.34	2.30 (1.50)	0.10–6.70	2.64 ± 1.51	2.30 (1.80)	0.50–9.30	0.76	213.8	0.448
Lymphocytes [thousand/µL]	1.93 ± 0.60	1.83 (0.66)	1.03–4.20	2.09 ± 0.60	2.07 (0.89)	1.01–4.06	2.165	219	0.031
Lymphocytes [%]	34.55 ± 8.17	33.10 (10.80)	19.10–56.40	34.24 ± 6.48	33.90 (8.90)	18.80–50.80	0.132	185	0.895
Neutrophils [thousand/µL]	3.09 ± 1.03	2.90 (1.19)	1.59–6.92	3.42 ± 1.21	3.20 (1.44)	1.59–8.04	2.154	219.9	0.032
Neutrophils [%]	54.19 ± 8.59	55.50 (10.60)	31.90–72.80	54.64 ± 7.19	55.00 (9.70)	32.20–72.10	0.104	193	0.917
PLT [thousand/µL]	251.69 ± 50.21	249.00 (67.00)	164.00–428.00	257.27 ± 56.83	249.00 (82.00)	104.00–394.00	0.658	224.5	0.511
MPV [fL]	10.83 ± 0.84	10.80 (1.40)	8.90–12.70	10.79 ± 0.84	10.70 (1.10)	9.00–13.60	−0.437	211	0.663
PDW [fL]	12.56 ± 1.70	12.40 (2.50)	9.00–16.90	12.54 ± 1.74	12.30 (2.20)	9.00–18.20	−0.189	211.8	0.851
P-LCR [%]	31.49 ± 7.15	31.30 (12.30)	15.50–46.50	31.18 ± 7.28	30.60 (9.90)	15.30–52.90	−0.333	212.7	0.74
PCT [%]	0.27 ± 0.05	0.27 (0.06)	0.17–0.40	0.28 ± 0.06	0.27 (0.08)	0.13–0.41	0.507	219.6	0.612

Hb—hemoglobin; RBC—red blood cells; Ht—hematocrit; PLT—platelet count; M—mean; SD—standard deviation; *n*—number of participants in the subgroups.

**Table 2:** Serum concentrations of selected biochemical parameters in the studied women according to the body mass index (BMI).

Parameter	BMI < 25 (*n* = 101)			BMI ≥ 25 (*n* = 129)			Brunner-Munzel Test	*df*	*p*
	M ± SD	Me (IQR)	Min–Max	M ± SD	Me (IQR)	Min–Max			
Glucose [mg/dL]	83.14 ± 9.47	82.50 (12.40)	62.8–105.5	87.29 ± 9.65	86.20 (12.70)	65.6–121.5	3.036	202	0.003
HbA1c [%]	5.33 ± 0.27	5.31 (0.32)	4.55–6.05	5.49 ± 0.34	5.41 (0.42)	4.78–7.04	3.699	221.5	<0.001
Insulin [µIU/mL]	7.32 ± 3.07	7.10 (3.40)	1.80–19.00	11.78 ± 6.57	9.80 (6.50)	3.20–35.80	7.418	227.9	<0.001
HOMA-IR	1.52 ± 0.71	1.47 (0.73)	0.30–4.01	2.58 ± 1.55	2.16 (1.58)	0.60–8.17	7.65	228	<0.001
QUICKI	0.37 ± 0.03	0.36 (0.03)	0.31–0.48	0.34 ± 0.03	0.34 (0.03)	0.28–0.42	−7.65	228	<0.001
TCh [mg/dL]	205.38 ± 36.13	205.20 (39.70)	124.7–346.3	209.76 ± 34.31	207.30 (51.50)	134.3–297.4	1.031	221.7	0.304
HDL [mg/dL]	70.98 ± 16.94	71.30 (23.60)	28.50–114.20	61.64 ± 14.66	61.40 (21.20)	32.70–106.60	−4.623	210.1	<0.001
LDL [mg/dL]	115.59 ± 29.61	115.24 (33.74)	44.46–211.16	123.56 ± 30.37	120.44 (40.84)	59.24–203.08	1.749	221.1	0.082
TG [mg/dL]	90.68 ± 43.19	84.50 (29.30)	37.80–378.40	121.51 ± 61.11	106.00 (58.30)	48.70–353.80	4.933	227.9	<0.001
Cortisol [µg/dL]	16.21 ± 7.60	15.02 (9.57)	5.39–45.80	14.62 ± 6.20	13.31 (6.28)	5.14–46.83	−1.513	191.8	0.132
Adropin [pg/mL]	709.92 ± 779.38	387.40 (642.90)	22.50–3898.67	810.92 ± 812.95	504.00 (756.70)	65.60–3876.00	1.727	198.8	0.086
Adiponectin [ng/mL]	12,717.30 ± 7184.87	11,746.30 (8630.40)	729.58–35,349.80	10,738.36 ± 5564.16	9723.30 (7061.80)	2389.20–26,768.50	−1.952	194.5	0.052
IDO [ng/mL]	29.64 ± 21.27	20.52 (31.20)	1.58–88.35	35.51 ± 23.50	27.08 (41.72)	6.03–89.98	2.077	214.8	0.039
Tryptophan [µg/mL]	13.10 ± 4.72	13.21 (5.44)	2.61–32.55	12.73 ± 4.97	12.56 (4.78)	4.31–34.64	−1.183	211.6	0.238
Kynurenine [ng/mL]	605.09 ± 154.23	607.81 (201.80)	248.22–1033.00	656.18 ± 195.62	619.70 (222.10)	177.40–1373.00	1.993	218.8	0.048
Serotonin [ng/mL]	195.60 ± 112.58	162.30 (127.40)	15.87–575.10	188.38 ± 113.07	171.51 (128.50)	6.13–765.80	−0.262	218.1	0.794
Visfatin [ng/mL]	4.89 ± 3.72	3.13 (4.54)	0.20–16.21	5.87 ± 4.02	4.71 (5.97)	0.66–16.48	1.934	218.9	0.054

M—mean; SD—standard deviation; *n*—number of participants in the subgroups; HbA1c—glycated hemoglobin; HOMA-IR—homeostasis model assessment of insulin resistance; QUICKI—quantitative insulin sensitivity check index; TCh—total cholesterol; HDL—high-density lipoprotein; LDL—low-density lipoprotein; TG—triglyceride; IDO—indoleamine 2.3-dioxygenase.

**Table 3:** Correlations between chemokines and metabolic/inflammatory biomarkers in women with normal and elevated BMI.

Marker	BMI < 25														
	CXCL1			CXCL2			CXCL3			CXCL5			CXCL9		
	r	*df*	*p*	r	*df*	*p*	r	*df*	*p*	r	*df*	*p*	r	*df*	*p*
Adropin [pg/mL]	0.007	99	0.947	−0.06	68	0.621	0.259	95	0.010 *	−0.046	68	0.705	−0.037	49	0.797
Adiponectin [ng/mL]	−0.052	99	0.609	−0.27	68	0.024 *	−0.216	95	0.033 *	−0.15	68	0.215	−0.229	49	0.107
IDO [ng/mL]	0.011	99	0.913	0.022	68	0.856	0.121	95	0.238	−0.079	68	0.518	0.022	49	0.877
Tryptophan [µg/mL]	−0.026	99	0.798	−0.014	68	0.909	−0.121	95	0.237	0.007	68	0.956	−0.116	49	0.419
Kynurenine [ng/mL]	0.122	99	0.226	0.022	68	0.855	0.236	95	0.020*	−0.013	68	0.917	−0.032	49	0.824
Serotonin [ng/mL]	−0.049	99	0.623	−0.08	68	0.511	−0.167	95	0.103	0.03	68	0.803	0.045	49	0.755
Visfatin [ng/mL]	0.034	99	0.736	0.049	68	0.690	0.03	95	0.768	−0.114	68	0.345	0.052	49	0.715
Marker	**BMI ≥ 25**														
	**CXCL1**			**CXCL2**			**CXCL3**			**CXCL5**			**CXCL9**		
	**r**	***df***	***p***	**r**	***df***	***p***	**r**	***df***	***p***	**r**	***df***	***p***	**r**	***df***	***p***
Adropin [pg/mL]	0.147	125	0.098	0.052	73	0.658	0.138	122	0.125	−0.092	66	0.455	−0.129	70	0.279
Adiponectin [ng/mL]	0.016	125	0.859	0.224	73	0.054	−0.015	122	0.866	0.08	66	0.517	−0.051	70	0.668
IDO [ng/mL]	0	125	0.997	0.042	73	0.723	−0.031	122	0.736	−0.066	66	0.594	−0.25	70	0.034 *
Tryptophan [µg/mL]	−0.016	125	0.858	0.25	73	0.030 *	−0.07	122	0.442	0.195	66	0.111	−0.127	70	0.288
Kynurenine [ng/mL]	−0.032	123	0.721	0.049	71	0.682	−0.009	120	0.922	0.014	64	0.911	−0.042	69	0.728
Serotonin [ng/mL]	−0.101	125	0.259	−0.099	73	0.399	−0.133	122	0.140	−0.138	66	0.261	−0.088	70	0.461
Visfatin [ng/mL]	−0.092	125	0.304	0.007	73	0.952	−0.094	122	0.297	−0.041	66	0.740	−0.243	70	0.040 *

IDO—indoleamine 2.3-dioxygenase, *r*—Pearson’s correlation coefficient, *df*—degrees of freedom, *p*—*p*-value. * *p*-values marked with an asterisk (*) were nominally significant at the 0.05 level, but did not remain significant after false discovery rate (FDR) correction for multiple testing (Benjamini–Hochberg method). These results should be interpreted with caution as potential false positives.

## Data Availability

The dataset is available on request from the authors.

## Supplementary Materials

The following supporting information can be downloaded at: https://www.mdpi.com/article/10.3390/nu17172885/s1, Table S1. Reference values; Table S2. Serum concentrations of selected hormonal parameters in women according to the body mass index (BMI); Table S3. Levels of inflammatory markers and immune response indices according to the body mass index (BMI); Table S4. Correlation between chemokine concentrations, proinflammatory cytokines, and inflammatory indices in women with normal and elevated BMI; Table S5. Correlations between the concentration of chemokines and the blood morphological parameters all studied women.
