# Supplementary material for: Systemic Inflammation Indices, Chemokines, and Metabolic Markers in Perimenopausal Women
Source: Nutrients. 2025 Sep 6;17(17):2885. doi: 10.3390/nu17172885 (PMC12430069; doi:10.3390/nu17172885)
Supplement: Supplementary file 1 [file nutrients-17-02885-s001.zip › nutrients-3835868-supplementary.pdf]

Supplementary materials

Table S1. Reference values

Blood count	Reference values
Hb [g/dl]	11,2-15,7
MCV [fl]	79,4-94,8
MCH [pg]	25,6-32,2
RBC [mln/µl]	3,93-5,22
Ht [%]	34,1-44,9
MCHC [g/dl]	32,2-35,5
RDW-CV [%]	11,7-14,4
Leukocytes [thousand/µl]	3,98-10,04
Monocytes [thousand/µl]	0,20-1,0
Monocytes [%]	2-10
Basophils [thousand/µl]	0,02-0,10
Basophils [%]	0,0-2,0
Eosinophils [thousand/µl]	0,03-0,27
Eosinophils [%]	1,0-6,00
Lymphocytes [thousand/µl]	1,0-3,0
Lymphocytes [%]	20-40
Neutrophils [thousand/µl]	2-7
Neutrophils [%]	40-80
PLT [thousand/µl]	150-400
MPV [fl]	9,40-12,5
PDW [%]	9,8-16,2
P-LCR [%]	19,1-46,6
PCT [%]	0,17-0,35
Glucose [mg/dl]	<100
HbA1C [%]	4-6 % (health) / 6.5% (with DM)
Insulin [µIU/ml]	<10
TC [mg/dl]	< 200

HDL [mg/dl]	>45
LDL [mg/dl]	< 115
TG [mg/dl]	<150
Cortisol [µg/dl]	5 -25
IL-1β [pg/ml]	No data
TNF-α [pg/ml]	< 16
IFN-γ [IU/ml]	<0,35
IL-6 [pg/ml]	< 1,8
IL-1α [pg/ml]	No data
CRP [mg/l]	<5,0

Table S2. Serum concentrations of selected hormonal parameters in women according to the body mass index (BMI).

**Parametr**	**BMI ≥ 25**				**BMI ≥ 25**				Brunner-Munzel Test	df	p
	**N**	**M ± SD**	**Me (IQR)**	**Min–Max**	**N**	**M ± SD**	**Me (IQR)**	**Min–Max**			
**CXCL1 (GRO)**	101	825.31 ± 1595.14	253.55 (236.03)	91.99–9474.52	127	781.65 ± 1263.29	287.66 (389.51)	87.85-7548.18	0.729	218.9	0.467
**CXCL2 (GRO)**	70	196.83 ± 301.56	57.27 (184.44)	27.03–1444.33	75	159.38 ± 228.96	73.75 (118.26)	27.03–1373.59	0.701	129.5	0.485
**CXCL3 (GRO)**	97	341.00 ± 875.25	105.16 (176.83)	37.79–7962.26	124	570.52 ± 1324.77	130.32 (208.89)	19.86–7719.80	1.44	207.3	0.151
**CXCL5 (GRO)**	70	2178.10 ± 2347.84	1183.16 (1142.82)	360.20–9994.93	68	2684.06 ± 2872.29	1515.25 (2551.50)	468.86–14274.92	1.486	135.7	0.140
**CXCL9 (MIG)**	51	448.56 ± 733.71	156.35 (362.19)	48.92–4099.87	72	495.39 ± 692.45	202.73 (329.26)	52.91–3480.46	1.247	93.9	0.216

M—mean.; SD—standard deviation.; *N* whole cohort size; *n*—number of participants in the subgroups.

Table S3. Levels of inflammatory markers and immune response indices according to the body mass index (BMI).

**Parameter**	**BMI ≥ 25**				**BMI ≥ 25**				Brunner-Munzel Test	df	*p*
	**N**	**M ± SD**	**Me (IQR)**	**Min–Max**	**N**	**M ± SD**	**Me (IQR)**	**Min–Max**			
**IL-1β**	101	189.12 ± 86.10	10.05 (363.85)	0.35-926.90	129	187.79 ± 280.81	19.42 (247.85)	0.35–998.30	0.428	212.4	0.669
**TNF-α**	81	6.36 ± 8.81	4.50 (5.30)	0.70–68.38	110	7.94 ± 11.70	4.84 (6.52)	0.70–81.10	1.045	169.4	0.298
**IFN-γ**	101	0.16 ± 0.73	0.03 (0.09)	0.03–7.31	129	0.21 ± 0.70	0.03 (0.11)	0.03–7.22	0.501	226.8	0.617
**IL-6**	101	31.23 ± 73.57	13.90 (20.16)	1.99–535.10	129	60.06 ± 134.70	19.52 (24.64)	1.99–787.80	2.107	219.2	0.036
**IL-1α**	101	3.50 ± 5.76	2.27 (0.83)	0.16–45.20	129	4.03 ± 9.70	2.35 (0.74)	0.33–78.90	0.573	190.1	0.567
**CRP**	101	1.44 ± 0.91	0.99 (0.52)	0.99–6.52	129	2.56 ± 2.50	1.74 (2.16)	0.99–17.90	5.987	228	<0.001
**SII**	101	430.46 ± 179.18	396.71 (279.50)	128.63–901.3	129	437.59 ± 178.86	391.27 (188.64)	105.00–990.0	0.179	191.3	0.858
**SIRI**	101	0.78 ± 0.39	0.72 (0.49)	0.25–1.87	129	0.80 ± 0.36	0.76 (0.44)	0.21–2.20	0.792	194.8	0.429

IL-1β – interleukin-1 beta; TNF-α – tumor necrosis factor alpha; IFN-γ – interferon gamma; IL-6 – interleukin-6; IL-1α – interleukin-1 alpha; CRP – C-reactive protein; SII – systemic immune-inflammation index.

Table S4. Correlation between chemokine concentrations, proinflammatory cytokines, and inflammatory indices in women with normal and elevated BMI.

**BMI < 25**															
**Marker**	**CXCL1**			**CXCL2**			**CXCL3**			**CXCL5**			**CXCL9**		
	**r**	**df**	***p***	**r**	**df**	***p***	**r**	**df**	***p***	**r**	**df**	***p***	**r**	**df**	***p***
IL-1β	–0.066	99	0.512	–0.012	68	0.921	–0.078	95	0.450	–0.108	68	0.375	0.233	49	0.099
TNF-α	0.018	79	0.875	0.176	56	0.187	0.072	-75	0.536	0.257	50	0.066	0.065	43	0.672
IFN-γ	–0.026	99	0.795	–0.041	68	0.736	0.024	95	0.816	–0.041	68	0.739	–0.079	49	0.583
IL-6	–0.057	99	0.571	–0.043	68	0.724	0.048	95	0.641	–0.065	68	0.592	0.090	49	0.529
IL-1α	–0.044	99	0.665	–0.040	-68	0.741	–0.057	-95	0.581	–0.076	68	0.533	–0.073	49	0.612
CRP	–0.057	99	0.574	0.027	68	0.824	–0.025	95	0.809	0.061	68	0.615	–0.017	49	0.907
SII	0.185	99	0.064	0.254	68	0.034*	–0.070	95	0.493	0.319	68	0.007*	0.245	49	0.083
SIRI	0.126	99	0.209	0.335	68	0.005*	–0.108	95	0.294	0.388	68	<0.001†	0.134	49	0.347

**BMI ≥ 25**															
**Marker**	**CXCL1**			**CXCL2**			**CXCL3**			**CXCL5**			**CXCL9**		
	r	df	*p*	r	df	*p*	r	df	*p*	r	df	*p*	r	df	*p*
IL-1β	–0.042	125	0.637	–0.090	73	0.444	–0.172	122	0.056	–0.120	66	0.332	–0.055	70	0.645
TNF-α	–0.106	106	0.276	–0.062	63	0.624	–0.118	105	0.227	–0.128	52	0.355	–0.156	61	0.223
IFN-γ	–0.061	125	0.497	–0.122	73	0.296	–0.051	122	0.576	–0.176	66	0.150	–0.103	70	0.387
IL-6	0.009	-125	0.919	0.044	73	0.711	0.097	122	0.286	0.150	66	0.222	0.017	70	0.886
IL-1α	–0.067	125	0.452	–0.045	73	0.704	–0.050	122	0.585	–0.070	66	0.569	0.378	70	0.001†
CRP	0.003	125	0.978	–0.090	73	0.442	0.045	122	0.617	–0.082	66	0.506	0.062	70	0.605
SII	0.088	125	0.324	–0.024	73	0.838	0.085	122	0.349	0.066	66	0.595	0.164	70	0.168
SIRI	0.010	125	0.908	–0.008	73	0.947	0.112	122	0.217	0.056	66	0.651	0.267	70	0.023*

r – Pearson's correlation coefficient, ***df*** – degrees of freedom, ***p*** – p-value.

* p-values marked with an asterisk (*) were nominally significant at the 0.05 level, but did not remain significant after false discovery rate (FDR) correction for multiple testing (Benjamini-Hochberg method). These results should be interpreted with caution as potential false positives.
† Remained significant after FDR correction.

Table S5. Correlations between the concentration of chemokines and the blood morphological parameters all studied women

	CXCL 1 (GRO) [pg/ml]			CXCL 2 (GRO) [pg/ml]			CXCL 3 (GRO) [pg/ml]			CXCL 5 [pg/ml]			CXCL 9 (MIG) [pg/ml]		
	r	df	p	r	df	p	r	df	p	r	df	p	r	df	p
**Hb [g/dl]**	-0.157	226	0.018*	-0.007	143	0.935	-0.096	219	0.154	-0.012	136	0.888	0.015	121	0.869
**MCV [fl]**	-0.188	226	0.004*	-0.129	143	0.122	-0.134	219	0.047*	-0.068	136	0.430	-0.237	121	0.008*
**MCH [pg]**	-0.201	226	0.002*	-0.131	143	0.117	-0.133	219	0.048*	-0.090	136	0.296	-0.218	121	0.015*
**RBC [mln/µl]**	0.027	226	0.685	0.125	143	0.136	0.020	219	0.766	0.070	136	0.412	0.221	121	0.014*
**Ht [%]**	-0.117	226	0.078	0.038	143	0.652	-0.069	219	0.305	0.043	136	0.619	0.051	121	0.576
**MCHC [g/dl]**	-0.165	226	0.013*	-0.084	143	0.315	-0.065	219	0.338	-0.075	136	0.383	-0.110	121	0.224
**RDW-CV [%]**	0.117	226	0.078	0.093	143	0.268	-0.001	219	0.992	-0.072	136	0.401	0.139	121	0.124
**Leukocytes [thousand/µl]**	-0.005	226	0.940	0.135	143	0.105	-0.036	219	0.596	0.040	136	0.643	0.248	121	0.006*
**Monocytes [thousand/µl]**	-0.024	226	0.72	0.084	143	0.315	-0.015	219	0.822	0.145	136	0.091	0.106	121	0.245
**Monocytes [%]**	0.061	226	0.363	-0.023	143	0.781	0.081	219	0.232	0.092	136	0.282	-0.097	121	0.287
**Basophils [thousand/µl]**	0.058	225	0.381	0.043	142	0.607	-0.089	218	0.187	-0.107	136	0.21	0.018	120	0.842
**Basophils [%]**	-0.033	226	0.615	-0.105	143	0.210	-0.101	219	0.134	-0.237	136	0.005*	-0.110	121	0.226
**Eosinophils [thousand/µl]**	-0.015	226	0.828	0.005	143	0.952	-0.025	219	0.711	0.105	136	0.22	-0.079	121	0.383
**Eosinophils [%]**	0.013	226	0.843	-0.083	143	0.323	-0.011	219	0.870	0.033	136	0.704	-0.076	121	0.405
**Lymphocytes [thousand/µl]**	-0.063	226	0.342	-0.003	143	0.972	-0.106	219	0.116	-0.073	136	0.393	0.037	121	0.688
**Lymphocytes [%]**	-0.085	226	0.199	-0.171	143	0.040*	-0.105	219	0.119	-0.144	136	0.093	-0.259	121	0.004*
**Neutrophils [thousand/µl]**	0.026	226	0.692	0.179	143	0.032*	0.012	219	0.855	0.082	136	0.341	0.307	121	$<0.001^{\dagger}$
**Neutrophils [%]**	0.065	226	0.329	0.169	143	0.042*	0.081	219	0.228	0.118	136	0.168	0.275	121	0.002*
**PLT [thousand/µl]**	0.129	226	0.052	0.093	143	0.265	0.102	219	0.130	0.016	136	0.855	0.085	121	0.350
**MPV [fl]**	0.013	226	0.839	0.087	143	0.295	-0.012	219	0.854	-0.055	136	0.525	0.078	121	0.390
**PDW [%]**	0.062	226	0.352	0.091	143	0.276	0.022	219	0.743	-0.024	136	0.781	0.139	121	0.125
**P-LCR [%]**	0.033	226	0.615	0.097	143	0.245	-0.004	219	0.954	-0.038	136	0.662	0.093	121	0.307
**PCT [%]**	0.148	226	0.026	0.135	143	0.106	0.106	219	0.117	-0.002	136	0.977	0.121	121	0.183
**Glucose [mg/dl]**	-0.102	226	0.126	-0.135	143	0.105	-0.100	219	0.138	-0.187	136	0.028	-0.157	121	0.083
**HbA1C [%]**	0.007	226	0.919	-0.168	143	0.043*	-0.043	219	0.525	-0.073	136	0.394	-0.055	121	0.543
**Insulin [µIU/ml]**	0.021	226	0.751	-0.073	143	0.383	0.026	219	0.703	-0.025	136	0.770	0.005	121	0.958

**HOMA-IR**	0.003	226	0.970	-0.086	143	0.301	0.007	219	0.913	-0.056	136	0.517	-0.020	121	0.825
**QUICKI**	0.001	226	0.989	0.093	143	0.267	-0.003	219	0.960	0.057	136	0.505	0.028	121	0.758
**TC [mg/dl]**	0.090	226	0.175	-0.026	143	0.755	0.024	219	0.726	0.058	136	0.498	-0.049	121	0.589
**HDL [mg/dl]**	0.021	226	0.751	-0.027	143	0.746	-0.062	219	0.362	0.070	136	0.416	-0.097	121	0.286
**LDL [mg/dl]**	0.084	226	0.207	0.007	143	0.932	0.050	219	0.463	0.012	136	0.893	-0.031	121	0.738
**TG [mg/dl]**	0.001	226	0.994	-0.060	143	0.473	0.017	219	0.807	-0.027	136	0.757	0.079	121	0.384
**Cortisol [µg/dl]**	0.036	226	0.587	0.000	143	0.998	-0.057	219	0.400	-0.010	136	0.910	-0.039	121	0.665

r – Pearson's correlation coefficient, df – degrees of freedom, p – p-value.

* p-values marked with an asterisk (*) were nominally significant at the 0.05 level, but did not remain significant after false discovery rate (FDR) correction for multiple testing (Benjamini-Hochberg method). These results should be interpreted with caution as potential false positives.

† Remained significant after FDR correction.
